# Cryoelectron Tomography of HIV-1 Envelope Spikes: Further Evidence for Tripod-Like Legs

**DOI:** 10.1371/journal.ppat.1000203

**Published:** 2008-11-14

**Authors:** Ping Zhu, Hanspeter Winkler, Elena Chertova, Kenneth A. Taylor, Kenneth H. Roux

**Affiliations:** 1 Department of Biological Science and Institute of Molecular Biophysics, Florida State University, Tallahassee, Florida, United States of America; 2 Center for Structural and Molecular Biology and National Laboratory of Biomacromolecules, Institute of Biophysics, Chinese Academy of Sciences, Chaoyang District, Beijing, China; 3 AIDS Vaccine Program, SAIC Frederick, National Cancer Institute at Frederick, Frederick, Maryland, United States of America; Harvard Medical School, United States of America

## Abstract

A detailed understanding of the morphology of the HIV-1 envelope (Env) spike is key to understanding viral pathogenesis and for informed vaccine design. We have previously presented a cryoelectron microscopic tomogram (cryoET) of the Env spikes on SIV virions. Several structural features were noted in the gp120 head and gp41 stalk regions. Perhaps most notable was the presence of three splayed legs projecting obliquely from the base of the spike head toward the viral membrane. Subsequently, a second 3D image of SIV spikes, also obtained by cryoET, was published by another group which featured a compact vertical stalk. We now report the cryoET analysis of HIV-1 virion-associated Env spikes using enhanced analytical cryoET procedures. More than 2,000 Env spike volumes were initially selected, aligned, and sorted into structural classes using algorithms that compensate for the “missing wedge” and do not impose any symmetry. The results show varying morphologies between structural classes: some classes showed trimers in the head domains; nearly all showed two or three legs, though unambiguous three-fold symmetry was not observed either in the heads or the legs. Subsequently, clearer evidence of trimeric head domains and three splayed legs emerged when head and leg volumes were independently aligned and classified. These data show that HIV-1, like SIV, also displays the tripod-like leg configuration, and, unexpectedly, shows considerable gp41 leg flexibility/heteromorphology. The tripod-like model for gp41 is consistent with, and helps explain, many of the unique biophysical and immunological features of this region.

## Author Summary

The envelope (Env) spikes on the surface of HIV-1 and SIV virions facilitate target cell tropism, binding, and entry, and serve as the sole targets of humoral (antibody-mediated) immunity. X-ray crystallography has previously revealed the atomic structures of key core domains and peptides of the gp120 and gp41 Env spike subunits, but the manner by which these components are arranged in the Env spike is still speculative. Cryoelectron tomography (cryoET) affords a view of the entire Env spike in the context of the intact virion. We have previously published a cryoET model of the SIV Env spike which showed a unique tripod-like leg configuration for the solvent-exposed (external) gp41 stalk region. This model is consistent with, and helps explain, many of the unique biophysical and immunological features of this region. Subsequently another group using similar technology and virions reported a spike model displaying a compact gp41 stalk inconsistent with our splayed-leg spike model. In this report, we apply enhanced analytical cryoET procedures to show that HIV-1 also displays the tripod-like leg configuration, and shows considerable gp41 leg flexibility/heteromorphology. These results have implications for the design of effective vaccines targeting this region and may provide new insights into Env spike function.

## Introduction

HIV-1 and the closely related SIV envelope (Env) spikes are composed of a trimer of heterodimers [1]–[6]. The base of the Env spike is comprised of three gp41 subunits, each of which possesses, from N-terminal to C-terminal, a fusion peptide, N-terminal heptad repeat, disulfide loop, C-terminal heptad repeat, membrane proximal external region (MPER), transmembrane domain, and cytoplasmic tail (CT). The relative positions of these various elements in the mature untriggered spike are largely unknown [7].

In contrast to gp41, the configuration of gp120 is better defined structurally. The CD4-liganded core structure consists of three subregions, the inner domain, the outer domain and the bridging sheet [8],[9]. The atomic structure of the unliganded SIV core has recently been described [10]. For both atomic structures, some of the more flexible elements, including V loops, N and C-terminal peptides and much of the glycan shield, were either deleted from the crystallization construct or were not resolvable due to flexibility [8]–[10].

The inherent flexibility of the V loops is a well recognized characteristic of HIV gp120 and has been suggested to be an important component of the viral defense against humoral immunity. Similarly, the CD4 binding site (CD4bs) components display flexibility, limiting the ability of most potential anti-CD4bs Abs to effectively bind, a process known as entropic masking [11].

Electron microscopy (EM) is an important adjunct to atomic structural studies and has the potential to allow the placement of the atomic structures of gp120 and gp41 core fragments and peptides, as well as the unresolved flexible components, into the global structural context of the Env spikes in situ [12]. Early work by Gelderblom and others showed virions covered with varying numbers of spikes [13]–[15]. A substantial fraction of purified spikes from HIV-1 and SIV were shown to display 3-fold symmetry though other forms were observed [1],[2]. By negative stain electron tomography, clear evidence for 3-fold symmetry was observed for a mutant form of SIV exhibiting Env with a truncated cytoplasmic tail [6]. The picture for HIV-1 is less clear in that the presumptive Env spikes appeared to display structural heterogeneity ([6] and unpublished data). Biochemical evidence for structural heterogeneity has also been published [1],[16],[17]. Because of the potential for morphological artifacts resulting from the use of the negative staining EM technique, including the attachment to a carbon substrate, pH changes, and drying, definitive analyses of the spike architecture could not be performed by this method [6].

More recently, we have further investigated the overall configuration of the SIV Env spike using cryoEM tomography (cryoET) wherein samples are preserved in a frozen hydrated state, free of the potential staining and drying artifacts common to negative staining [18]. Advantage was taken of the high level of Env spike incorporation and expression on the short-tailed mutants (SIVmac239/251 tail CEMx 174) (∼70 spikes/virion vs. ∼7–10 for wtSIV and wtHIV-1) thus aiding in data collection for the cryoET studies. HIV-1 variants with comparably high levels of Env spike expression are not available. The results from the SIV mutant show an Env spike in which each protomer of the presumptive trimeric gp120 head displays several morphological features and the solvent-accessible portion of gp41 forms a tripod-like set of legs. We were able to provide a tentative fit for the unliganded SIV gp120 core atomic structure [10] within the cryoET density volume and suggested that regions of unoccupied volume represented the masses of the V1/V2 and V3 loops missing from the atomic structure.

In an effort to determine the degree of structural heterogeneity within the spike population, the individual spike volumes were subjected to classification analysis in which the spikes were sorted into groups according to structural similarity. The results showed that most of the spikes were similar in form [18].

Subsequently, Zanetti et al. published a cryoET study showing an SIV Env spike average which differed from Zhu et al. in several important aspects [19]. For example, each gp120 subunit consisted of a simple globular mass adequate in volume to accommodate the atomic model of the core structure [10] but not large enough for the considerable mass of the V1/V2 and V3 loops. They also reported that the gp41 ectodomain formed a compact stalk rather than the flared tripod configuration that we observed. These differences are not easily reconciled since both groups took advantage of similar SIV short-tailed mutant virus.

In this report we have extend our cryoET analysis of the Env spike structure to include native (unmutated) Env spikes on wtHIV-1 and have now applied enhanced data collection and analysis techniques to generate 3D models. The data reveal that, as with our model of the short tailed SIV Env spike mutant, the wtHIV-1 displays tripod-like gp41 “legs”, at least in a significant percentage of the spikes. However, application of new approaches to search for structurally distinct morphological variations (i.e., a new classification algorithm) within the data suggests considerable conformational variability which likely reflects a more flexible structure than previously described.

## Materials and Methods

### Viruses

The highly purified virus (HIV-1 BaL / SUPT1-CCR5 CL.30, lot p3955) used in this study was produced and provided by the AIDS Vaccine Program, SAIC Frederick, Inc., NCI, Frederick, MD. The production and purification procedures were as previously described [20]. The samples were treated with 2,2′-dithiodipyridine (Aldrithiol-2, AT-2), a process that eliminates viral infectivity while preserving Env structure and function [20],[21].

### Cryo EM preparation

Fifteen µl of AT-2-treated viruses (∼2.8 mg/ml total protein) were added to 120 µl of PBS and pelleted at 25 psi for 15 min in an Airfuge centrifuge (Beckman Coulter) equipped with an A100/30 rotor. The pellets were resuspended in 10 µl of PBS of which 3.5 µl was placed on a 300 mesh R2/1 Quantifoil grid (Quantifoil, Jena, Germany) for 1 min. Excess virus and buffer was blotted with filter paper. The grid was then rapidly vitrified by plunging into liquid ethane in a liquid nitrogen bath using a homemade plunging apparatus.

### Cryo electron tomography

The EM grids were transferred to a Gatan 626 cryoholder (Gatan, Pleasanton, CA) and examined under low dose conditions on a Philips (FEI, Eindhoven, Netherlands) CM300-FEG microscope operated at 300 kV. Single axis tilt series were recorded at 43,200× magnification using a Tietz Tem-Cam F224 slow scan CCD camera (2,048×2,048 pixels, Tietz Video, Gauting, Germany) and associated EM-MENU software. The pixel size at the specimen level is 5.56Å. Each tilt series consisted of 70–80 images recorded over an angular range of ±60° to ±70° at increments chosen according to the cosine rule [22]. The electron dose was estimated at 1–2 e^−^/Å^2^ per image.

### Image analysis

#### General methods

The projection images, collected at different tilt angles, were aligned based on the cross-correlation method using the PROTOMO program package [23]. Tomograms were computed by weighted back-projection and the individual spike volumes were picked and subject to a repeated cycle of 3D alignment and classification using algorithms as described below.

The raw tomograms were computed from uniaxis tilt series and therefore have resolution anisotropy due to the missing wedge. Potential effects of the missing wedge were compensated with improved image analysis procedures. The subvolume alignment used constrained correlation [24] [Winkler et al., *J. Struct. Bio.*, in press], and averaging was carried out in Fourier space, which facilitated the exclusion of the missing regions in the summation of structure factors. Additionally, neither the spike volume nor the references were subject to any symmetry imposition during the alignment.

The center of each virus particle and the positions of each Env spike were manually determined by visual inspection, and a volume (64×64×64 pixels) encompassing each spike and associated viral membrane were windowed for analysis. We intentionally selected spikes from the entire surface of the virus including the tops and bottoms as well as the sides of the virions. We judge that the spikes selected from the tops/bottoms give better views of the oligomeric structure of the spikes even though in this orientation, the membrane is not visible. Spikes selected from the sides provide the information on the membrane position with respect to the heads.

**Figure 1:**
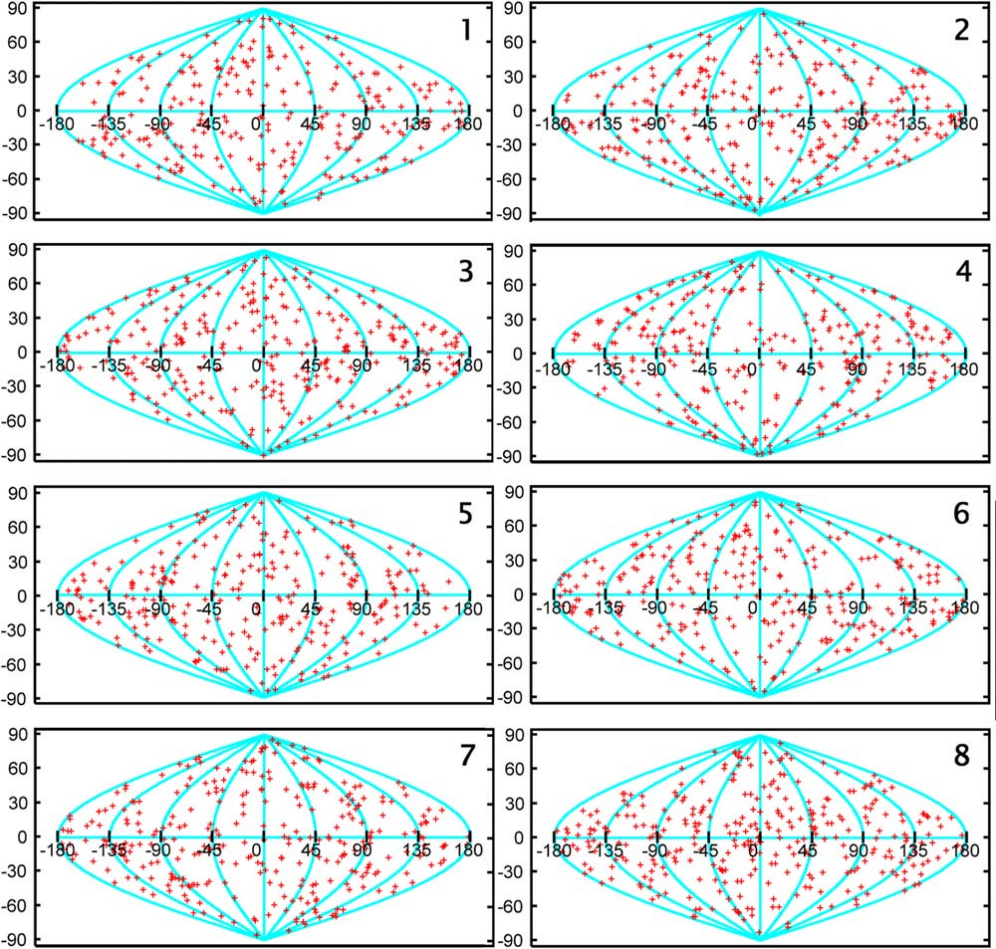
Spatial distribution of the HIV-1 Env spikes. The selected (whole) HIV-1 spikes were aligned and sorted into eight classes. For each class, the tilt axis direction of each spike was calculated with respect to the spike coordinate frame and mapped onto the surface of a unit sphere (red “+” symbols), which was then converted to a two-dimensional representation with a sinusoidal projection. Latitude 90 degrees (vertical coordinate), for instance, corresponds to a tilt axis direction along the spike axis pointing to the spike head, −90 degrees to the spike base. The sinusoidal projection preserves the area, so that the density of the plotted points is the same as on the spherical surface. Numbers in the upper right corner of each panel correspond to the class numbers illustrated in Figure 3A.

The initial spike orientations (spike axes) were calculated as follows. First, the equation of an ellipsoid was derived from the coordinates of the picked spikes by a least squares fit. Then the normal vectors on the surface of the ellipsoid were computed at the spike positions. These normal vectors were used as the initial estimate for the spike axis, thereby minimizing the angular range that must be searched for refined alignment. Each spike volume was then transformed based on these vectors so that they and their associated membranes were roughly aligned. A global average (without imposed symmetry) was generated and used as an initial reference to align the raw spike volumes. Because spikes are distributed over the entire surface of virions and are randomly oriented with respect to each other in the specimen, the initial average had no regions that are data poor due to the missing wedge. To align the direction of the spike axes more accurately, a rotational orientation search was carried out within a cone of half-width 15° and using a step size of 2°. In the initial cycle, no rotational alignment about the spike axis was included in the search. In subsequent cycles, the half-width was reduced to 4° and then 2° in the directional search, and an additional rotational search about the spike axis was introduced, which used a step size in the range from 6° to 3°. The aligned volumes were then classified into ∼10–20 classes using hierarchical ascendant methods [25]. Class averages were generated and were used as the multiple references for subsequent alignment cycles. All spike volumes were subjected to repeated cycles of multi-reference alignment and classification, until the appearance of the classes no longer changed. In the final cycle, spike volumes were classified into eight classes and their density averages generated. During the alignment step, some density due to the membrane is included within the windowed region. The global distribution of the spikes for each of the 8 final subclasses derived from the entire spikes volumes is shown in Figure 1.

#### Independent analyses of spike heads and legs

**Figure 2:**
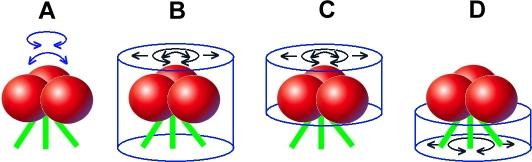
Schematic representation of the alignment and classification schemes applied to selected spike volumes. Red spheres and green lines represent gp120 and gp41 subunits, respectively. (A) Possible modes of flexibility within any given Env spike (blue arrows). Cylindrical masks (blue) encompass the entire spike (B), the gp120 head region (C), or the gp41 leg region (D). Black arrows indicate translocation, rotation, and tilting applied during alignment.

We used multivariate data analysis [25] to investigate the structural variability of the whole Env spike, and the head (gp120) and leg (gp41) regions separately as illustrated in Figure 2B–2D. A binary mask was constructed to select the voxels that are specifically used in the analysis. Note that the terms “mask” and “window” as used here are related but not identical. The process of windowing regions of the subvolumes for alignment adds an apodized edge to the boundary to avoid generating Fourier ripples in the cross correlation function and to minimize the contributions of unrelated image voxels (which are essentially solvent derived) from impacting the correlation peak height. The mask used for multivariate data analysis is strictly binary; the voxels selected all have the same weight and there is no apodization.

For analyses of the whole spikes, the classification mask was cylindrical with a diameter of 14 nm that enclosed the entire spike and legs but specifically excluded the membrane. When the membrane is included, the spike subvolumes segregate into top-bottom and side views irrespective of other features. For the independent head and leg analysis, this cylinder was divided into two non overlapping regions enclosing the separate features. The following alignment and classification schemes were evaluated: (1) alignment and classification of the whole spike, masked as described above, (2) alignment of the whole spike, classification based on the head or leg region only, which was achieved by modifying the classification mask appropriately; and (3) both alignment and classification using only the head or leg region. The alignment scheme in method (3) was different in that the alignment was used as a refinement subsequent to the alignment based on a whole spike, only one alignment cycle was carried out, only the change in the polar angle was used and the membrane densities were windowed out in the alignment. Although classifications were done based on the classification mask, averaging was always done on complete subvolumes.

#### Volume model generation to evaluate levels of inherent symmetry

To assess the relative symmetry and structural commonality revealed by each of the alignment and classification masking schemes, we selected those classes that were subjectively judged to display the most obvious tendency toward three-fold symmetry within each masking strategy set and subjected them to alignment and averaging into a single class. In practice, this meant that the most symmetric 3 to 6 class averages of the original eight class averages resulting from each classification scheme were mutually aligned and averaged to yield an “idealized” class average. No masks were applied to the class averages generated for visualization. The resulting maps were displayed using Chimera software to show an average spike rendered as a volume surface.

For display purposes, three-fold symmetry was imposed only at this final step. Surface rendering models were then produced from both the unsymmetrized and symmetrized density maps wherein the optimally aligned heads (leg masked) and legs (head masked) were digitally grafted together.

## Results/Discussion

In the absence of crystallized Env trimers, the analysis of intact virion-expressed Env spikes by cryoET may represent the best approach to determining important structural features of Env on the native virus. However, the use of cryoET for determining macromolecular conformations *in situ* is a rapidly evolving technique that has yet to reach its full potential and for which there is no generally agreed upon set of procedures. Our results and those of Zanetti et al. [19] have provided a first approximation of the overall structure of SIV Env. It was therefore of some concern that both attempts produced somewhat different results. It has been suggested [26] that the use, by the two groups, of a structure with imposed rotationally symmetry as an early reference for aligning the raw spike subvolumes and the subsequent application of 3-fold symmetry to the final image average may have generated or overly enhanced symmetry in the previously published SIV spike reconstructions [18],[19] It has also been suggested that our choice to ignore the consequences of a missing wedge of information resulting from the inherent inability to capture images of the virions over a full 180° tilt range may have led to incomplete data collection and artifactual distortion of the spike model. The latter point is probably not as great an issue as implied since data collected separately from the spikes projecting from the sides of the virions and from spikes projecting from the tops and bottoms of the virions gave similar results; any missing wedge effects would have been expected to differentially distort the images along different axes in the two sets [18].

To address these concerns, we have now used a missing wedge compensated multi-reference alignment and averaging scheme [Winkler et al., *J. Struct. Bio.*, in press] to analyze wtHIV-1 Env spikes which addresses the problems of reference bias and incomplete data. In addition, we have investigated the potential advantages of applying sorting and classification schemes not only to the entire spike volume but also, independently, to the key head and leg subregions of the spike volumes. This latter approach would be expected to generate an improved average if molecular motions of the heads and the legs are uncoupled to a significant degree.

### Overall spike morphology

**Figure 3:**
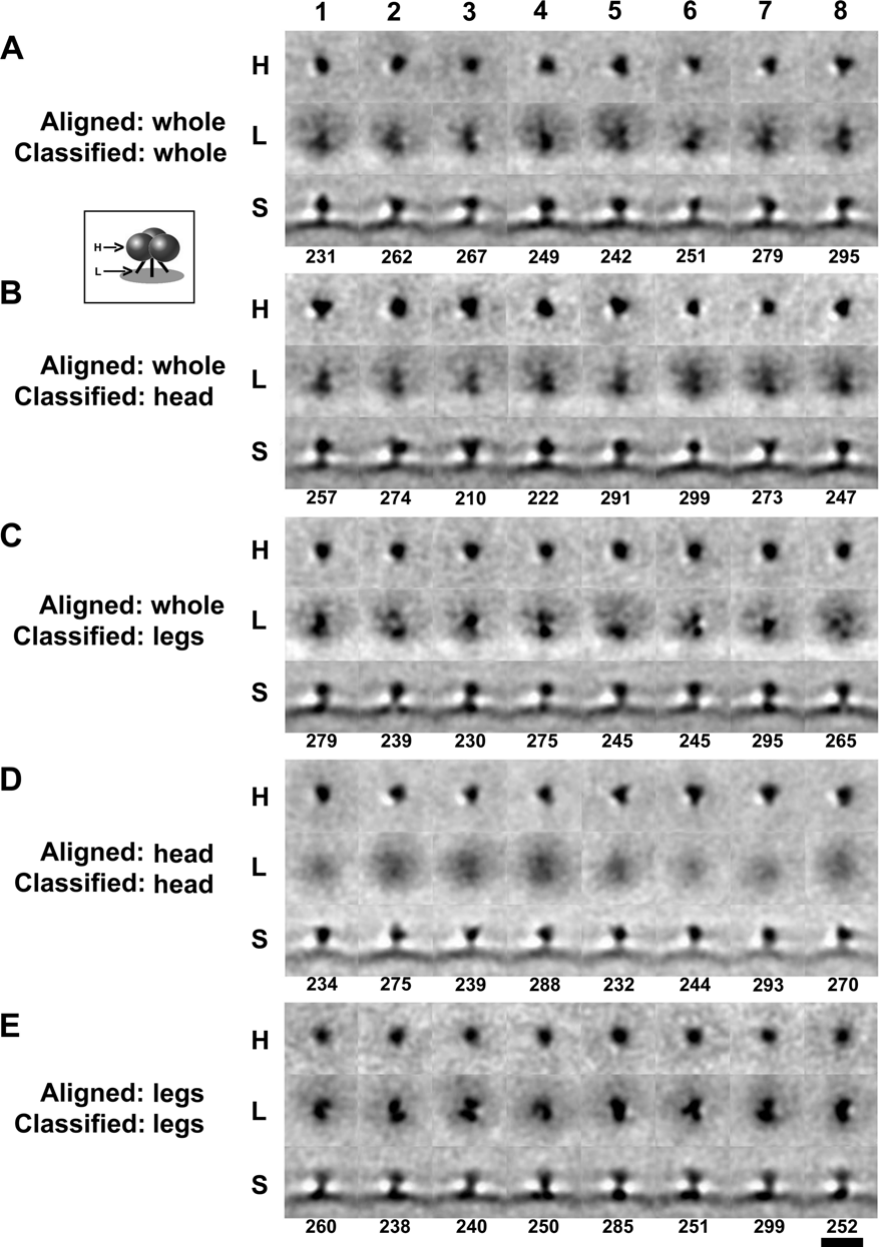
Representative digital transverse and longitudinal sections of the aligned and classified HIV-1 Env spikes. The various alignment and classification combinations are indicated (A–E) and refer back to the mask depictions in Figure 2. Eight classes were produced for each combination (1–8) and the numbers of individual spikes volumes in each class is indicated below the panels. For each exercise, ∼800 spikes were automatically discarded as not fitting any of the eight classes. The boxed insert depicts the approximate locations of the sections shown (H = transverse section through the head, L = transverse section through the legs, S = longitudinal section showing side view of the spike (above) and viral membrane (below)). Bar = 20 nm.

Our initial selection of 2,874 Env spike volumes, derived from 181 wt HIV-1 virions was subsequently reduced to 2,070 through programmatic elimination of lower quality selected volumes during the automated phase of the classification process. Subsequently, the spikes were sorted into eight classes based on multivariate data analysis and the class members in each were aligned and averaged. The surface distribution of the selected spikes in the entire set as well as in each of the eight classes suggests that the sampling was random and that the classification scheme did not result in the biased clustering of positionally discrete subpopulations (e.g., top/bottom- or side-arrayed) as might be expected with inadequate missing wedge compensation (Figure 1). Figure 3A shows cross-sections through the broadest portion of the head (H), the leg region (L) just above the membrane (see boxed insert for illustration), and a side view (S) of a section parallel to the axial plane with the membrane at the bottom of each of the eight classes. Although heteromorphic, each class average displayed overall dimensions similar to each other (∼12 nm high, ∼11 nm wide) and to our previously published SIV spike model (13.7 nm high, 10.5 nm wide) [18], thus increasing our confidence that the selected viral surface molecules represent bona fide Env spikes. Four of the classes showed a tendency toward 3-fold symmetry (judged visually), at least in the head region (Figure 3A, H5–8) while the other four showed asymmetry, though none were circular or shapeless. A comparative analysis of a cross-section through the base of each spike just above the membrane showed heterogeneity in structure incompatible with the single gp41 stalk model [19]. Most classes show two (Figure 3A, L3, 6, 8) or three densities (Figure 3A, L1, 2, 5, 7), with some showing additional diffuse signals. None, however, show three truly distinct leg densities.

Subvolume classification schemes can give a sense of the range of structure variation in the subvolumes selected for comparison. The real strength of the approach is that it facilitates a determination as to whether subsets (classes) of objects comprise the larger population. Relatively rigid, structurally distinct subpopulations, if present and significantly different from one another, should sort into distinct classes when subjected to multivariate data analysis. Even an inherently flexible structure displaying more random movement over significant distances would be expected to sort into separate classes although the result would tend to be blurred compared to the result expected in the case of structurally discrete states. Conformational variability *within* individual Env subunits (i.e., gp120 protomers) would not be detectable at the current resolution limits of this technique. Evidence of inter-subunit conformational variability should be most easily discerned by examining the spikes down the polar axis, i.e., screening for symmetry by viewing from above or below.

Even greater modes of flexibility might be manifested as twisting and/or rocking motion of gp120 and gp41 with respect to one another (Figure 2A). Because the alignment algorithms assess voxel density (signal intensity), the entire mass of the spike contributes to the placement of any given spike density within the average. Because the gp120 head is of greater mass than the solvent exposed regions of gp41, i.e., the MPER, the head would be expected to have a proportionally greater influence on the average. As a consequence, if the gp41 legs are in a tripod-like configuration but some were twisted, for example, with respect to the gp120 heads, the gp41 signals would be blurred to yield a cone of weak density rather than discrete, dense tripod-like legs in the averaged models. Similarly, the gp41 and membrane density might also impede alignment of the gp120 heads should the head and legs be twisted or bent with respect to one another. This is, in fact, what we observed for the MPER when the various classes, representing all of the classified Env spikes, were averaged together (data not shown).

To test this hypothesis, we reasoned that if the gp41 leg/membrane regions and gp120 head regions were independently aligned and/or classified, the weighting effect of one region upon the other would be eliminated and structural subregion classes with more discrete structural characteristics might emerge. Figure 2B–2D depicts the various head and leg subvolume alignment and classification schemes used to address this issue. For simplicity, the membrane is not depicted in the diagram. Alignments and, independently, classifications were based on the density data within either the whole spike, head, or leg/membrane regions.

Overall, the data show that subunit alignment and classification enhanced the symmetry of the targeted subregion and conversely, had the predictable effect of blurring the detail of the subregion excluded from the classification. For example, when the gp120 head was excluded, allowing the leg densities to drive the classification (Figure 3C), the gp41 leg region showed 3 of 8 classes (Figure 3C, L4, 6, 8) (representing 38% of total spikes) with three more-or-less distinct densities (judged subjectively) with a tendency toward 3-fold symmetry, a pattern that was less obvious following alignment and classification of the whole spike where only 1 of 8 classes (11% of total spikes) showed this pattern (Figure 3A, L1). Surprisingly, when both alignment and classification were driven by the leg densities, one class showed clear 3-fold leg symmetry (Figure 3E, L6, 12%) though all the others showed multi-leg asymmetry. In some classes where two legs are obvious, one of the legs appears extra thick (Figure 3C, L1, 2, 3; 3E, L 4, 7) possibly indicating a (transient?) association of two of the three legs.

When the gp41 leg region was excluded from classification, 5 of 8 classes (representing 63% of total spikes) had gp120 head regions that displayed a tendency toward 3-fold symmetry (Figure 3B, H1, 3, 5, 6, 8) compared to 4 of 8 classes (51% of total spikes) for whole spike alignment and classification (Figure 3A, H5–8). This trend was more pronounced when both alignment and classification were performed using just the head volumes (Figure 3D) wherein 6 of 8 classes (75%) were trimer-like (Figure 3D 3–8). Thus, even though there was clearly a tendency toward symmetry within the spikes, it was less evident following whole spike averaging, even when whole spike classification (into 8 classes) was applied. Interestingly, subvolume classification appeared about as effective in enhancing applicable head or tail images irrespective of whether the alignment was based on the whole spike or the targeted subvolume of the spike (compare Figure 3B to 3D and 3C to 3E). Stated another way, the use of subvolume *alignment* appeared less important to the final outcome than the application of subvolume *classification*. The reason for the less dominant effect in enhancing substructures could be attributed to the fact that selective alignment based on head or leg regions was carried out as a refinement of the already aligned whole spikes. Thus, the applied incremental changes do not appear as significant as the structural differences obtained by classification.

A significant percentage of the classified spikes and spike components deviated considerably from 3-fold symmetry. The reasons for this are unclear but include bona fide segmental flexibility/heteromorphology, and “noisy” data, a general characteristic of cryoEM data where contrast is inherently low. It is worth noting that in neither SIV nor HIV-1 did we observe any evidence of conserved structural features immediately below the membrane as would have been expected if the CT of Env were rigid or associated with a geometrically arrayed submembrane matrix layer.

**Figure 4:**
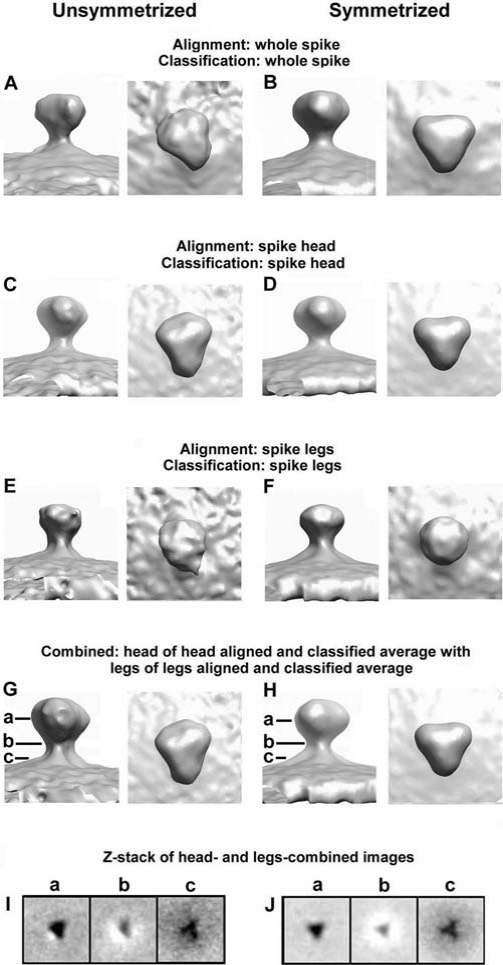
Surface rendered models of averaged selected classes. Alignment and classification combinations as presented in Figure 2 and described in the text. The left (A,C,E) and right (B,D,F) panel pairs show the averaged spikes before (left) and after (right) symmetrization. Within each pair are side (left) and top (right) views. Panels (G) (unsymmetrized) and (H) (symmetrized) represent idealized chimeric models composed to the head regions of (C) and (D) grafted onto the legs of (E) and (F), respectively. (I) and (J) represent transverse digital sections through the head (a), waist (b), and membrane-proximal leg region (c) as indicated in (G) and (H).

In order to construct a single volume rendering of an “idealized” HIV spike, we selected the spike subregion classes showing the most symmetric features and averaged them together as single classes. These averaged classes were then displayed as surface renderings as illustrated in Figure 4. For example, the head-aligned/head-classified classes represented by Figure 3D (3–8) were averaged as a single class (Figure 4C) and, in the final step, 3-fold symmetrized (Figure 4D). The best leg-aligned/leg-classified classes (from Figure 3E, 3, 6, 7) were similarly combined, aligned, and averaged (Figure 4E and F). The optimized heads from the first set of models (Figure 4C and 4D) were then grafted onto the legs of the second set of models (Figure 4E and 4F) to yield the composite HIV-1 spikes shown in Figure 4G and 4H. To determine the correct rotational orientation of the legs with respect to the head, we averaged the most symmetric classes from the whole unmasked classification scheme and measured the rotational orientation of the legs with respect to the head (data not shown). Figure 4I and 4J represent transverse digital sections through the unsymmetrized and symmetrized chimeric models in Figure 4G and 4H, respectively. The ‘a’, ‘b’ and ‘c’ designations represent the head, midsection, and membrane-proximal leg sections, respectively.

A comparison of the HIV-1 composite model (Figure 4G and 4H) to our previously published SIV model [18] shows protein masses comparable to the main and lateral lobes, a less well defined peak, but no mass corresponding to the proximal lobe (Figure 4C). Unlike the Zanetti et al. model, we find no discernable cavity at the head-leg interface [19]. Our HIV-1 model appears to have three splayed legs though they are less well defined compared to our SIV model [18]. The radii of the legs in those HIV-1 classes where three discrete legs were visible were comparable to that previously reported for SIV (∼4.8 nm) [18]. No legs were seen in the Zanetti et al. model [19].

Evidence for Env spike structural heterogeneity also comes from a reanalysis of the Zhu et al. SIV data [Winkler et al., *J. Struct. Bio.*, in press]. In contrast to what was originally reported [18], heteromorphology in the spike appearance and apparent flexibility are also seen in that data when subjected to the same general alignment and classification scheme reported here. Although, not subjected to independent targeted head and leg classification and reassembly, trimeric structures in the SIV head and the splayed leg conformations were also seen. Thus, the methods used here have generated similar results on two independent data sets. We now feel that both the Zanetti et al. [19] and Zhu et al. 2006 [18] models were unduly influenced by the reference that was selected in the earliest cycle and that this reference accentuated certain features at the expense of others. Consequently, the details of the respective density maps and fitting of the atomic core structures in those reports should be considered as provisional.

### Significance of tripod-like legs

There is a general view that one of the main reasons no suitable Env-based vaccine for the induction of effective humoral protection has been developed relates to the difficulty in engineering soluble versions that faithfully mimic the viral spike surface configuration (reviewed in [27],[28]). Early monomeric constructs largely failed due to the exposure of immunodominant epitopes on the non-neutralizing face, a region believed buried in the gp120 subunit interface in the oligomer. Consequently, numerous attempts have been made at generating trimeric soluble constructs. However, many such constructs have proven inherently unstable with unacceptably high levels of subunit dissociation and/or aggregation. Strategies to circumvent this obstacle include mutational disruption of the protease cleavage sites between gp120 and gp41, inter-subunit disulfide bonding and other stabilization enhancing point mutations, and the addition of trimerization motifs (reviewed in [27],[28]). These approaches have had varying degrees of success at stabilizing the trimer and occluding the non-neutralizing face but have yet to faithfully mimic the antigenic profile of authentic membrane-associated trimers.

Our initial observation that the MPER of Env gp41 appeared to be in an open tripod-like conformation rather than in the traditionally-depicted compact stalk configuration provided a plausible explanation for the failure of at least some engineered version of Env trimers to adopt the native configuration [18]. Specifically, the trimerization motifs used to date, which bunched the C termini of the MPER tightly together, might force the MPERs into an unnatural configuration, perhaps altering and/or weakening the already inherently unstable interactions between the rest of the tripartite subunits. The resultant constructs may thus behave more like three tethered monomers rather than true trimers. On the other hand, if, as our data indicates, the Env spike is, to a degree, polymorphic and/or displays considerable component flexibility, a fully rigidified recombinant Env spike may not mimic the structure of virion-associated Env either. However, it may well turn out that rigid constructs, even if they don't fully mimic natural virus-associated Env might nevertheless serve as more effective vaccines. The production of a crystal structure of an Env trimer in its (near) native configuration would significantly advance our understanding of these and other issues relating to Env, however the prospects of success are diminished if the variations in form we observe are the result of segmental flexibility.

Several reasons for moving cautiously in fully embracing the tripod-legged paradigm have been put forth [12],[26]. First, the original modeled cryoET Env spike was derived from 3D tomograms of SIV rather than HIV-1. Although the structure of the Env spikes on the two AIDS viruses have been assumed to be structurally similar and the atomic structures of the gp120 of the unliganded HIV-1 and liganded SIV core proteins have been extensively compared [10],[29],[30], true similarity at the atomic level has yet to be formally demonstrated. Indeed, our previous negative stain EM tomogram studies have found HIV-1 spikes to be less uniformly configured than those on SIV ([6] and unreported data). Other data indicate that the degree of compactness and subunit accessibility to ligand binding varies significantly between SIV and HIV-1 and even between different strains of each [11], [28], [31]–[33]. Second, the cryoET-modeled SIV Env spike derives from a mutated version of SIV displaying a truncated CT. While this feature enhanced the expression of Env spikes on virions, thus facilitating data collection, it could be argued that the loss of a considerable segment of CT might well influence Env spike structure, especially in the most closely associated MPER [34]–[36]. This concern is somewhat ameliorated by data demonstrating that the Env spikes on these mutants are sufficiently functional so as to support efficient viral fusion and host cell infection [37]. Third, as described above, it has been argued that our previous data collection and processing schemes might have skewed the data and thus the model. Fourth and finally, Zanetti et al. [19], analyzed a short-tailed SIV virion nearly identical to those used by us yet they generated a rather different Env spike average model. Differences were observed both in the head (more compact in Zanetti et al.) and the presumptive gp41 solvent exposed region (compact vertical stalk in Zanetti et al.). Some of the potential reasons and technical issues relating to these differences have been discussed elsewhere [12],[26].

The data reported here support one of the key findings of our previous spike model in that we again find evidence of tripod morphology in the MPER. More importantly, this feature is now extended to include Env spikes from non-mutated wtHIV-1. To allay concerns about artificially enhanced symmetry, no symmetric references were utilized nor was enforcement of three-fold symmetry applied in the alignment or classification schemes used to generate the eight classes. Yet evidence pointing to a tendency toward 3-fold symmetry both in the gp120 head region and in the gp41 MPER emerged, at least in some of the class averages. Even in those classes without three leg masses, typically two masses are present as is diffuse additional density, a pattern more consistent with a three flexible leg model than a compact stalk model. Only in the final surface rendered models of averaged selected classes was symmetry enforced (Figure 4B, 4D, 4F, 4H).

The accumulating evidence regarding the biophysical features of both the MPER and the neutralizing MAbs that target this region are consistent with it having extensive membrane association [38]–[44] (see [45] and [46] for a comprehensive reviews of the MPER). This region may also be fairly flexible. For example, the segment encompassed by the 4F10, Z13e1 and 2F5 epitopes may transition between alpha-helical and alternative motifs to allow exposure of key residues that would otherwise be on opposite sides of the presumed alpha helical structure of this region. Such a transition would be required for effective binding of these MAbs in a membrane-associated environment [43],[45]. Recent high resolution NMR evidence suggested that the HIV-1 4E10 targeted epitope of the MPER is initially largely buried in the lipid bilayer and may be partially extracted upon 4E10 binding [42]. The conformational change associated with this interaction is facilitated by a flexible hinge-like region within the epitope. Such inherent flexibility may well contribute to our observed heteromorphology in the leg region and is consistent with the spread tripod-like leg orientation in a significant fraction of the Env spikes.

### Note of added information

During the manuscript review process, Liu et al. 2008 [47] published a cryoET model of the HIV-1 spike with features that differed from the both the Zhu et al. [18] and Zanetti et al. [19] SIV spike as well as those reported here in several respects. Liu et al. report a compact stalk for gp41 and a Z-axis-elongated structure for gp120 in which the monomeric subunits make minimal contact with each other. Their unliganded gp120 structure could not readily accommodate the unliganded core structure of Chen et al. [10] but was fitted instead with the CD4 liganded core structure [8],[9]. After evidence of symmetry became apparent in the early rounds of alignment, symmetry was imposed on subsequent rounds and spike densities not fitting this pattern were discarded. We suspect that this model, like those of Zhu et al [18] and Zanetti et al. [19], may be unduly influenced by reference bias and imposed symmetry.
